# Vitamin D Level Trajectories of Adolescent Patients with Anorexia Nervosa at Inpatient Admission, during Treatment, and at One Year Follow Up: Association with Depressive Symptoms

**DOI:** 10.3390/nu13072356

**Published:** 2021-07-09

**Authors:** Manuel Föcker, Nina Timmesfeld, Judith Bühlmeier, Denise Zwanziger, Dagmar Führer, Corinna Grasemann, Stefan Ehrlich, Karin Egberts, Christian Fleischhaker, Christoph Wewetzer, Ida Wessing, Jochen Seitz, Beate Herpertz-Dahlmann, Johannes Hebebrand, Lars Libuda

**Affiliations:** 1 Department of Child and Adolescent Psychiatry, University Hospital Münster, 48149 Münster, Germany; ida.wessing@ukmuenster.de; 2 Department of Medical Informatics, Biometry and Epidemiology, Ruhr-University Bochum, 44780 Bochum, Germany; timmesfeld@amib.ruhr-uni-bochum.de; 3 Department of Child and Adolescent Psychiatry, University Hospital Essen, University of Duisburg-Essen, 45147 Essen, Germany; judith.buehlmeier@uni-due.de (J.B.); johannes.hebebrand@uni-due.de (J.H.); lars.libuda@uni-paderborn.de (L.L.); 4 Department of Endocrinology, Diabetes and Metabolism, Division of Laboratory Research, University Hospital Essen, University of Duisburg-Essen, 45147 Essen, Germany; Denise.Zwanziger@uk-essen.de (D.Z.); Dagmar.Fuehrer-Sakel@uk-essen.de (D.F.); 5 Department of Pediatrics, Division of Rare Diseases, St Josef-Hospital, and CeSER, Ruhr-University Bochum, 44791 Bochum, Germany; corinna.grasemann@ruhr-uni-bochum.de; 6 Department of Child and Adolescent Psychiatry & Division of Psychological & Social Medicine and Developmental Neurosciences, Faculty of Medicine, Technische Universität Dresden, 01307 Dresden, Germany; Stefan.Ehrlich@uniklinikum-dresden.de; 7 Department of Child and Adolescent Psychiatry, Psychosomatics and Psychotherapy, University Hospital Wuerzburg, 97080 Wuerzburg, Germany; Egberts_K@ukw.de; 8 Department of Child and Adolescent Psychiatry and Psychotherapy, University Medical Center Freiburg, 79104 Freiburg, Germany; christian.fleischhaker@uniklinik-freiburg.de; 9 Department of Child and Adolescent Psychiatry and Psychotherapy, Kliniken der Stadt Köln, 51067 Cologne, Germany; WewetzerC@kliniken-koeln.de; 10 Clinic for Child and Adolescent Psychiatry, Psychotherapy and Psychosomatics, University Hospital RWTH Aachen, 52074 Aachen, Germany; jseitz@ukaachen.de (J.S.); bherpertz@ukaachen.de (B.H.-D.); 11 Faculty of Natural Sciences, Institute of Nutrition, Paderborn University, Consumption and Health, 33098 Paderborn, Germany

**Keywords:** vitamin D, supplements, anorexia nervosa, depressive symptoms, adolescents

## Abstract

(1) Background: Evidence has accumulated that patients with anorexia nervosa (AN) are at higher risk for vitamin D deficiency than healthy controls. In epidemiologic studies, low 25(OH) vitamin D (25(OH)D) levels were associated with depression. This study analyzed the relationship between 25(OH)D serum levels in adolescent patients and AN and depressive symptoms over the course of treatment. (2) Methods: 25(OH)D levels and depressive symptoms were analyzed in 93 adolescent (in-)patients with AN from the Anorexia Nervosa Day patient versus Inpatient (ANDI) multicenter trial at clinic admission, discharge, and 1 year follow up. Mixed regression models were used to analyze the relationship between 25(OH)D levels and depressive symptoms assessed by the Beck Depression Inventory (BDI-II). (3) Results: Although mean 25(OH)D levels constantly remained in recommended ranges (≥50 nmol/L) during AN treatment, levels decreased from (in)patient admission to 1 year follow up. Levels of 25(OH)D were neither cross-sectionally, prospectively, nor longitudinally associated with the BDI-II score. (4) Conclusions: This study did not confirm that 25(OH)D levels are associated with depressive symptoms in patients with AN. However, increasing risks of vitamin D deficiency over the course of AN treatment indicate that clinicians should monitor 25(OH)D levels.

## 1. Introduction

Anorexia nervosa (AN) is a complex disorder with a variety of comorbidities. Besides several other biological and psychological phenomena, depression and vitamin D deficiency are often documented in patients with AN [1].

Findings from a meta-analysis of observational studies until 2013 indicate that vitamin D levels in patients with anorexia nervosa (AN) are lower compared to healthy controls even when vitamin D intake appears equal [2]. Other studies have reported a lower prevalence rate of vitamin D deficiency in patients with AN than in the general US population. In the study of Mehler et al., only 30% of a total of 1026 patients with AN were reported to be deficient in vitamin D [3]. Hanachi et al. found a prevalence of 54% in 374 patients [4] (both cutoff 25(OH)D < 30 ng/mL (75 nmol/L)). In comparison, the prevalence of vitamin D deficiency in the total US population was above 64% from 1988 to 2010 [5]. Also, in two small Swedish AN samples, vitamin D deficiency was not even confirmed [6,7]. Aside from differences in study sample characteristics and laboratory analyses, the missing consideration of vitamin D supplementation as confounding variable in the statistical models probably explains these conflicting findings, since vitamin D supplementation is known to normalize vitamin D levels in patients with AN [2].

First and foremost, supplementation in case of vitamin D deficiency could be important for bone health during AN treatment, as a sufficient 25(OH)D level was found to be a key factor to increase bone mineral density in patients with AN [8,9]. However, there is increasing evidence from previous population-based cross-sectional and longitudinal studies that vitamin D deficiency is not only associated with bone health, but also with mental disorders such as major depression or depressive symptoms [10,11,12,13].

Within the ALSPAC cohort, 25(OH)D levels at the age of 10 years were inversely associated with self-rated depressive symptoms at 14 years (*n* = 2752) after adjustment for potential confounders (e.g., ethnicity, age, and gender) [11]. Depressive symptoms were assessed using the Mood and Feelings Questionnaire. Similarly, we showed an inverse association between 25(OH)D levels and the subscales emotional problems, peer relationship problems, and the total difficulties score of the Strengths and Difficulties Questionnaire in a representative sample of German children and adolescents (KIGGS study) [12].

Vitamin D supplementation studies in adults, however, revealed conflicting results on depression as shown by systematic reviews and meta-analyses of RCTs. The lack of effect of vitamin D supplementation on depression was explained by so-called “biological flaws” such as the lack of vitamin D deficiency in participants at baseline and an insufficient dose of vitamin D [14]. It was recommended that future RCTs should focus on individuals who are both depressed and vitamin D deficient [15]. In adolescents, such a RCT in day- and inpatient psychiatric treatment recently failed to show a vitamin D supplementation effect on self-rated depression, but parent-rated depression decreased more in the vitamin D group at the end of our study compared to the control group [16]. However, this RCT did not focus on patients with AN.

As major depression was the most frequent comorbidity in adolescents with AN in the large Anorexia Nervosa Day patient versus Inpatient (ANDI) multicenter trial [17], potential antidepressant effects of vitamin D supplementation could also be relevant for AN treatment. Thus, the aim of this analysis of the ANDI sample was to investigate in adolescent patients with AN whether:

1. patients with AN are at risk of 25(OH)D deficiency over the course of AN treatment;
2. supplementation of vitamin D during (in-)patient treatment reduces the risk of vitamin D deficiency at discharge;
3. 25(OH)D levels are inversely associated with depressive symptoms.

## 2. Materials and Methods

### 2.1. Study Sample

Ninety-three participants of the multicenter ANDI trial aged 11–18 years with complete data at admission (T0), discharge (T1), and one year after admission (T2) were included in the analysis [18]. All patients fulfilled DSM-IV criteria for AN [19] according to the Structured Interview for Anorectic and Bulimic Disorders (SIAB) for DSM-IV [20].

The detailed study design was recently described by Herpertz-Dahlmann et al. [18]. In brief, ANDI was conducted as a multicenter RCT including female patients with AN from five university clinics and one major hospital in Germany. The safety and efficacy of day patient treatment compared to inpatient treatment of AN were primary study aims. The participants underwent a three-week stepped care program of inpatient treatment to treat somatic and psychological consequences of starvation. After this treatment period, patients were randomized into one of two intervention groups, which included either continued inpatient (treatment as usual) or day patient treatment. The same multimodal treatment program was implemented in both settings (inpatient and day patient), which included the following interventions: weight restoration (a minimum weight gain of 300 g/week was targeted), individual and group nutritional counseling, cognitive–behavioral individual and group therapy, family therapy, and a group psychoeducation program for parents. Dietary treatment started with a reduced number of calories adapted to individual capacities with a minimum of 600 kcal/d, which was continuously increased in steps of 200 kcal/d until a steady weight gain of at least 500 g/d was reached. Diets were arranged individually with a specialized nutritionist and were in line with the German Society for Nutritions (DGE) guidelines for nutritional content [21].

Patients were discharged after a 2-week weight maintenance period (approximately the 15th–20th age-adjusted percentile). For the present analysis, data sets of both intervention groups were analyzed jointly (see Table 1 for the sample characteristics).

The study was conducted according to the guidelines of the Declaration of Helsinki and approved by the Ethics Committee of the University Hospital of the Technical University of Aachen (EK127/06, 30 October 2006). Informed written consent was obtained by patients and their legal guardians.

### 2.2. Data Assessments

All assessments considered in this analysis were assessed during admission (T0), discharge (T1), and one year follow up (T2).

The Beck Depression Inventory-II (BDI-II, German version) [22], which is a self-reported questionnaire, was used for the assessment of depressive symptoms. The BDI-II includes 21 items based on the DSM-IV diagnostic criteria for MDD. Higher scores on the four-point Likert scale (0–3 points) indicate a greater degree of depression. Thereby the BDI-II asks about the participant’s symptoms within the preceding two weeks.

Blood samples for 25(OH) vitamin D analysis in serum were drawn after an overnight fast in the morning and within the same week as anthropometric measurements at admission, discharge, and one year follow up. Blood serum samples were frozen at −80 °C and thawed for post-hoc analyzes of 25(OH)D levels in this study. 25(OH)D levels were measured by the Siemens ADVIA Centaur® Immunoassay System (Siemens Healthineers, Erlangen, Germany) at the central laboratory of the University Hospital Essen. The competitive immunoassay is a standardized laboratory measurement of 25(OH)D according to the NIST (National Institute of Standards and Technology, Erlangen, Germany). The intra-assay variation was <11.9%, the inter-assay variation was <5.3%, the functional sensitivity was 4.2 ng/mL (10.5 nmol/L), and the limit of detection was 3.2 ng/mL (8.0 mmol/L) according to the product insert. To be consistent with the terminology used in previous studies, we use the term “vitamin D deficiency”, although our cutoff of concentrations <20 ng/mL (50 nmol/L) was originally defined as “at risk for inadequacy” by the Institute of Medicine (IoM) [23]. Information on vitamin D supplementation during treatment were obtained from medical records-based prescription of vitamin D-containing drugs.

Anthropometric assessments comprised measurements of weight (in underwear) and height, which were used for calculation of BMI. Age- and sex-independent BMI percentiles and standard deviation scores (BMI-SDS) were calculated considering German reference data [24].

### 2.3. Statistical Analysis

Analysis of the data was conducted with “R” (www.r-project.org, version 3.5.1 accessed on 10 September 2021). For all analyses, two-sided tests were used, and *p*-values < 0.05 were considered significant.

Serum 25(OH)D over the course of AN treatment (T0, T1, and T2) was tested by ANOVA for repeated measurements. A logistic regression model was used to examine the impact of vitamin D supplementation during AN treatment on the risk of vitamin D deficiency at discharge. Vitamin D deficiency at admission and season were included as covariates.

In order to examine cross-sectional relationships between 25(OH)D levels and BDI-II scores, Pearson correlations were calculated for each measuring time point. Linear mixed-effects regression models, including both fixed and random effects, were used to construct a longitudinal model of depressive symptom trajectories between T0 and T2. Patient effect was modeled as random effect (random intercept per patient). As fixed effect, the model included 25(OH)D levels at T0, the interaction between 25(OH)D levels at T0 and time (T0, T1, or T2), and the individual change in 25(OH)D levels. The latter was calculated by subtracting baseline 25(OH)D levels from the respective 25(OH)D level at each time point. Accordingly, the model yielded 3 regression coefficients representing the following: (1) cross-sectional estimate—an estimate for the regression of 25(OH)D levels at baseline on depressive symptoms at baseline; (2) prospective estimate—the slope of the regression of 25(OH)D levels at baseline on the change in depressive symptoms among T0, T1 and T2; and (3) concurrent estimate—the slope of the regression of change in 25(OH)D levels among T0, T1, and T2 on the concurrent change in depressive symptoms. BMI-SDS at T0, the interaction between BMI-SDS at baseline and time, the change in BMI-SDS, age at T0, AN subtype (i.e., restrictive or binge-purge), use of antidepressants during inpatient treatment, duration of illness, and measuring time (T0, T1, T2) were included as covariates in the model. This approach of longitudinal data analysis to examine the association between the individual change in an exposure and concurrent changes in an outcome has been proposed to possess features of a quasiexperimental design [25].

## 3. Results

### 3.1. Study Sample Characteristics

At admission (T0), the mean age of the patients with AN was 15.3 (1.5) years, and the vast majority was of the restricting subtype (Table 1). Mean BMI was 15.1 (± 1.3) kg/m^2^ (BMI percentile 1.9 (± 3.2)), which increased over the course of AN treatment. Thirty percent of the patients showed a vitamin D deficiency at admission. This prevalence increased, especially after discharge, to 45% at 1 year follow up. Approximately half of the sample received vitamin D supplementation during treatment. Nearly one-third of the sample had a comorbid diagnosis of an affective disorder. BDI-II score was highest at admission (mean = 19.8, SD = 10.1) and halved at discharge (mean = 10.7, SD = 8.6). The included sample did not differ from the non-included sample (*N* = 79) of the ANDI study with respect to age, illness duration, body weight and diagnostic subtypes, or depressive symptoms at admission. The number of supplemented study participants was slightly higher in the non-included sample (68% vs. 54%, supplementary Table S1), but this difference was not significant (*p* = 0.073).

### 3.2. Trajectories of 25(OH)D Levels and Associations with Depressive Symptoms

Results from ANOVA revealed decreasing 25(OH)D levels over the course of AN treatment (*p* = 0.025) with pairwise comparisons showing significant decreases between T0 and T2 (ß = −5.3 (−10.0; −0.5); *p* = 0.027). Logistic regression showed that missing vitamin D supplementation was associated with higher risk of vitamin D deficiency at discharge (OR = 9.5, 95%CI (3.1, 34.7), *p* < 0.001).

**Figure 1:**
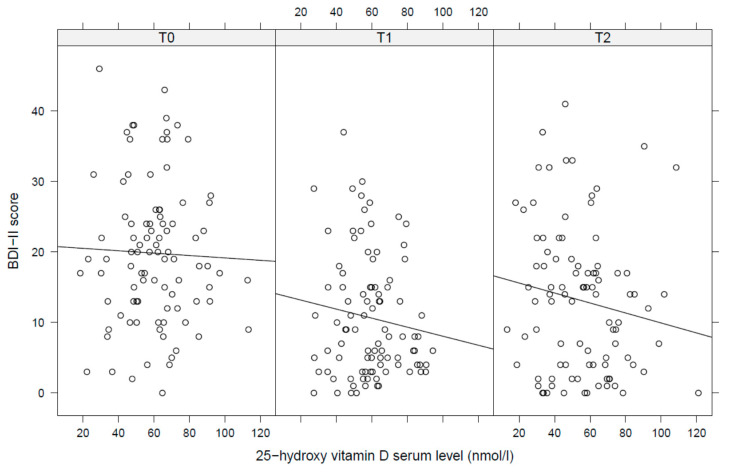
Pearson correlations between 25-hydroxy vitamin D serum levels and depressive symptoms (T0 = admission, T1 = discharge, T2 = 1 year follow up). BDI = Beck Depression Inventory, 25(OH)D = 25-hydroxy vitamin D serum level.

Pearson correlations between 25(OH)D level and BDI-II showed a negative, but non-significant association for each time point with slightly increasing correlation coefficients at later time points (Figure 1). Mixed linear regression analysis confirmed findings from these univariate models: 25(OH)D levels were neither cross-sectionally nor prospectively or longitudinally associated with BDI-II scores (Table 2). A diagnosis of the binge-purge subtype and the use of antidepressants was associated with a significantly increased BDI in this model (both *p* = 0.004).

## 4. Discussion

To our knowledge, this is the first study that reports trajectories of 25(OH)D levels in a relatively large adolescent sample of patients with AN over the course of (in-)patient treatment and after 1 year. The association of 25(OH)D levels with depressive symptoms was analyzed cross-sectionally, prospectively, and longitudinally. Three main findings can be summarized:

1. Vitamin D deficiency (<50 nmol/L) was found in only 30% of patients with AN at admission, but 25(OH)D levels decreased until 1 year follow up. Approximately 50% of the patients were vitamin D deficient after 1 year follow up (T1).
2. Vitamin D supplementation during treatment was associated with a lower risk of vitamin D deficiency at discharge.
3. 25(OH)D levels were not associated with depressive symptoms, neither cross-sectionally, prospectively, nor longitudinally.

Our results show that Vitamin D deficiency according to the cutoff level given by the Institute of Medicine (IoM) classifying 25(OH)D levels <20 ng/mL (50 nmol/L) is not necessarily a general problem in adolescent patients with AN in Germany at inpatient admission. The meta-analysis of Veronese and colleagues previously also showed that 25(OH)D levels in subjects with AN can be in the normal range, at least when vitamin D is supplemented [2]. Despite an unfavorable sun angle as a consequence of latitude, two studies from northern countries [6,7] reported median 25(OH)D levels in the adequate range in mixed samples of adolescent and adult patients. Carlsson and colleagues reported median values of 25(OH)D levels in patients with AN and healthy controls of 81 nmol/L (22–165 ng/mL) and 72 nmol/L (25–105 ng/mL), respectively [6]. In line with this, median serum 25(OH)D levels of 25 Swedish patients with AN were within the adequate range (84 nmol/L at baseline, 76 nmol/L after 12 weeks of inpatient weight gain) [7]. Vitamin D deficiency (<20 ng/mL) was present in 24% of 24 male patients with AN [9]. The majority of our sample also showed 25(OH)D values within the recommended range; at admission, only 30% of the patients were below the cutoff value of 50 nmol/L. Although different approaches of vitamin D analyses have to be taken into account, the prevalence of vitamin D deficiency in our study sample seems to be lower compared to the German child and adolescent population represented by the KIGGS sample [26]: on average, 50% of the adolescents from this study were below 50 nmol/L. Controversial results in the current literature may depend on widely diverging definitions of vitamin D deficiency [27]. The following cutoff values were used in the aforementioned studies: <20 nmol/L (8 ng/mL) [6,7], <50 nmol/L (20 ng/mL) [9], and <75 nmol/L (30 ng/mL) [3,4]. In the absence of an internationally consented threshold of optimal vitamin D status, several thresholds (<30, <40, <50, and <75 nmol/L) were used in the study of Schleicher and colleagues for prevalence analyses based on the US population [5].

One factor contributing to the comparatively high percentage of adequate vitamin D levels might be higher levels of physical activity of patients with AN (e.g., doing sports outside) [28]. Unfortunately, we did not assess outside activities within the ANDI trial. Furthermore, the history of vitamin D supplementation prior to clinical admission was not assessed, but this, we assume, may be higher compared to healthy adolescents, as a higher acceptance for dietary supplements have already been shown for girls with AN compared to healthy adolescents [29]. A further explanation could be that decreasing body fat in patients with AN leads to a stronger mobilization of vitamin D from adipose tissue. Several studies reported an inverse association between body fat and 25(OH)D levels. Both obese adults and children had lower 25(OH)D than healthy weighted controls [30,31,32]. It is important to mention that the present study group consists only of first-episode adolescent female patients. Thus, the divergent findings might result from different age groups and illness durations of the study samples. Adult patients with longer duration of illness might have a higher risk of vitamin D deficiency than adolescent patients. This hypothesis is supported by those studies that included only adolescent female patients [6,7]. During the (in-)patient treatment process from admission to discharge, 25(OH)D levels did not change in the patients of the ANDI study. In contrast, in the study of Svedlund and colleagues, a significant decrease of 25(OH)D levels was reported after 12 weeks of treatment [7]. Although different increments in body fat might contribute to these differing findings, the comparatively high frequency of vitamin D supplementation might be to a large extent responsible for the stable 25(OH)D level during treatment in our study: every second patient with AN received prescriptions or recommendations of vitamin D supplements from the involved physicians [33]. After one year follow up, 25(OH)D levels decreased compared to T0/T1. At this time point, patients were no longer under such a close medical observation as during inpatient or day patient care. Thus, the decrease of 25(OH)D after 1 year follow up could be explained by an insufficient vitamin D intake and/or a lack of supplementation. Additionally, the season of measurement time points (increased rate of patients measured during spring, see Table 1) and slight increases of BMI are potential contributors to the decreased 25(OH)D levels after 1 year follow up.

Our analyses did not reveal cross-sectional, prospective, or longitudinal associations of 25(OH)D levels and depressive symptoms in patients with AN. These results are in contradiction to other findings from observational studies in healthy children and adolescents [11,12], which show a significant inverse association between emotional/depressive symptoms and 25(OH)D levels. Although there is growing evidence for a relationship of 25(OH)D levels and mental health both in adults and in children, especially in terms of depression [10,13,34,35,36], findings of RCTs and Mendelian randomization analyses are conflicting and raise concerns on causality of vitamin D deficiency for depression [37,38]. Low 25(OH)D levels may reflect “ill health” in general, limiting vitamin D’s causal role for disease etiology [39].

Concerning our analyses, missing associations could result from adequate 25(OH)D levels in most patients, although masking their poor general health. Based on the BDI, patients showed mild or minimal depression. Depressive symptoms were most pronounced at the acute stage of starvation and decreased after weight gain at discharge. Thus, the severity of depression in patients at the different time points was probably too weak to detect associations. It was an interesting finding that correlations between 25(OH)D serum levels and depressive symptoms were constantly negative at each time point, which is in line with results from observational studies in children from the general population [11,12]. Accordingly, we cannot exclude that a study of a larger sample size could reveal significant associations in patients with AN. However, considering the missing significance of an association between 25(OH)D serum levels and depressive symptoms in our study, we assume that there might be other, more important etiological factors during the state of starvation responsible for depressive symptoms. Current complex etiological models include hormonal, metabolic, immunological, nutrition-based, microbiome-based, and genetic factors together with brain morphological changes [40,41,42,43]. Specifically, hypercortisolemia and hypoleptinemia have been reported to be associated with depressive symptoms in patients with AN [44,45,46,47,48,49]. Finally, patients with AN are at risk for multiple nutrients deficiencies [4]: zinc, copper, selenium, vitamin B1, vitamin B12, and vitamin B9, which could also contribute to depression. One may assume that multiple starvation-induced changes and specific nutrient deficiencies, inadequate vitamin D status being one, create clinical occurrence of depressive symptoms. In this situation, focusing on only one nutrient might be insufficient.

Considering the results from our study, the question remains whether vitamin D should be supplemented in patients with AN. On the one hand, this study did not reveal clear indications for routine vitamin D supplementation, since the prevalence of vitamin D deficiency was lower than in the general population and we failed to find any association with depressive symptoms of our patients. On the other hand, mean 25(OH)D levels decreased between admission and 1 year follow up, and vitamin D supplementation at admission substantially reduced the risk of vitamin D deficiency in our sample. Considering the fact that hypovitaminosis D during realimentation counteracts the favorable effects of refeeding with regard to bone health [8,50] it might be concluded that 25(OH)D levels should be constantly monitored over the course of AN treatment, and in case of deficiency, vitamin D should be supplemented.

### Limitations

To the best of our knowledge, this is the first longitudinal study in adolescents addressing the association between changes in 25(OH)D and concurrent changes in depressive symptoms. The mid-term follow up of this large study sample, the repeated measurements of patients with AN, and the available information represent a good opportunity to address the question of vitamin D’s influence on depressive comorbidities in patients with AN. However, as the study was not conceptualized for this specific question, some important information was lacking, e.g., detailed data on vitamin D supplementation for the time before admission and after discharge and information on supplementation dosing and duration during treatment.

As information on further important confounders like sun exposure and outside activity was also not available, the results might be biased and underestimate vitamin D’s effects on depressive symptoms. With regard to the generalizability of the study, it has to be noted that the mean duration of illness of the adolescent study sample was less than one year. Accordingly, this study only allows conclusions on the potential role of vitamin D at an early stage of AN.

BDI-II is a self-assessment tool, which is commonly used in depression research [51]. The sole use of self-reports, however, is critically discussed for assessment of depressive symptoms in adolescents [52,53]. Self- and parental-rated mental health brought only moderate correlations. Differences between informants increased with age in particular for internalizing problems. Independent clinician ratings might be more reliable for assessment of dietary effects on mental health in clinical adolescent samples in general [16].

Further studies should include more detailed information about supplements and consider additional confounders with regard to vitamin D status. Additionally, RCTs using vitamin D supplements focusing on AN patients with vitamin D deficiency and concurrent diagnosis of an affective disorder at baseline are needed. These should include clinician-based ratings of depressive symptoms.

## 5. Conclusions

Compared to healthy adolescents, vitamin D deficiency was less prevalent in patients with AN in Germany. Vitamin D supplements were frequently described, probably as a consequence of concerns by the responsible medical staff. However, against this background, 25(OH)D levels were not associated with depressive symptoms. Whether this is a simple result of adequate vitamin D status in the study sample or whether other pathophysiological factors have a stronger impact on depressive symptoms in patients with AN in general remains open. Nevertheless, with respect to the indisputable role of vitamin D for bone health, 25(OH)D levels should be monitored in patients with AN during the realimentation process considering increasing risks for vitamin D deficiency after discharge.

## Figures and Tables

**Table 1:** Sample characteristics of the patients (*N* = 93) with AN at respective measuring time points. Values are means ± SD or *N* (%) as indicated.

Characteristics at Inpatient Admission			
Age (years)	15.3 (±1.5)		
Duration of illness (weeks)	47.3 (±36.3)		
Premorbid BMI kg/m^2^	20.2 (±2.8)		
AN-subtype (*N* (%))			
Restrictive	79 (85%)		
Binge-Purge	14 (15%)		
Diagnosis of affective disorder (*N* (%)) *	25 (31%)		
Vitamin D supplementation (*N* (%))	50 (54%)		
Characteristics During Treatment			
	T0	T1	T2
BMI (kg/m^2^)	15.1 (±1.3)	18.2 (±0.9)	18.4 (±1.9)
BMI percentile	1.9 (±3.2)	19.3 (±7.9)	20.5 (±20.8)
BMI-SDS	−2.67 (±0.91)	−0.93 (±0.38)	−1.04 (±0.83)
BDI-II score	19.8 (±10.1)	10.7 (±8.6)	13.1 (±10.4)
25(OH)D (nmol/L)	60.3 (±19.0)	59.2 (±16.2)	55.1 (±21.6)
Vitamin D deficiency (<20 ng/mL (50 nmol/L)) (*N* (%))	28 (30%)	27 (29%)	42 (45%)
Season at measuring time point (*N* (%)):			
Winter (Nov–Mar)	37 (40%)	34 (37%)	34 (37%)
Summer (Jul–Oct)	28 (30%)	40 (43%)	28 (30%)
Spring (Apr–Jun)	28 (30%)	19 (20%)	31 (33%)
Antidepressants		10 (10.8%)	18 (19.4%)

T0 = admission, T1 = discharge, T2 = one year follow up after admission, BDI-II = Beck Depression Inventory, 25(OH)D = 25-hydroxy vitamin D serum level; AN = anorexia nervosa; * information available for 81 patients, SDS = Standard Deviation Score.

**Table 2:** Results from mixed linear models on cross-sectional, prospective, and longitudinal associations between serum 25(OH) vitamin D levels and depressive symptoms.

	Outcome: BDI-II Score at Each Measuring Time Point (T0, T1, T2)		
	ß	95% CI	*p*
Intercept	7.45	−9.80, 24.71	0.412
*25(OH)D estimates*			
Cross-sectional: 25(OH)D T0	−0.02	−0.12, 0.08	0.682
Prospective: 25(OH)D at T0 on change in BDI between T0 × T1	−0.05	−0.18, 0.07	0.388
Prospective: 25(OH)D at T0 on change in BDI between T0 × T2	−0.02	−0.14, 0.09	0.684
Longitudinal: Change of 25(OH)D on concurrent change in BDI	−0.04	−0.11, 0.04	0.385
*Covariates*			
Measuring time point: T1	−2.15	−12.71, 8.38	0.695
Measuring time point: T2	−3.27	−13.63, 6.96	0.541
Age	0.91	−0.16, 1.99	0.109
AN Subtype (Binge–Purge)	6.28	2.27, 10.30	0.004
Antidepressant usage	5.50	1.87, 9.29	0.004
Duration of illness	−0.02	−0.05, 0.02	0.462
BMI-SDS T0	0.23	−1.84, 2.30	0.833
Prospective: BMI-SDS at T0 on change in BDI between T0 × T1	1.49	−1.37, 4.35	0.318
Prospective: BMI-SDS at T0 on change in BDI between T0 × T2	1.09	−1.68, 3.84	0.45
Longitudinal: Change of BMI-SDS on concurrent change in BDI	−0.22	−2.32, 1.90	0.845

BDI = Beck Depression Inventory, 25(OH)D = 25-hydroxy vitamin D serum level.

## Data Availability

The data presented in this study are available on request from the corresponding author. The data are not publicly available due to ethical restrictions.

## Supplementary Materials

The following are available online at https://www.mdpi.com/article/10.3390/nu13072356/s1, Table S1: Sample characteristics of non-included (*N* = 93) and included (*N* = 79) patients with AN from the total ANDI study sample at in-/daypatient admission.
