# Supplementary material for: Vitamin D Level Trajectories of Adolescent Patients with Anorexia Nervosa at Inpatient Admission, during Treatment, and at One Year Follow Up: Association with Depressive Symptoms
Source: Nutrients. 2021 Jul 9;13(7):2356. doi: 10.3390/nu13072356 (PMC8308738; doi:10.3390/nu13072356)
Supplement: Supplementary file 1 [file nutrients-13-02356-s001.zip › nutrients-1259868-supplementary.pdf]

Supplementary Table 1. *Sample characteristics of non-included (N=93) and included (N=79) patients with AN from the total ANDI study sample at in-/daypatient admission. Values are means ± SD or N (%) as indicated.*

Total "ANDI" Sample	**Non-included**	**Included**	**p-value**
N=172	***N=79***	***N=93***	
Age	15.1 (1.58)	15.3 (1.45)	0.2167
Duration of illness (weeks)	48.7 (37.6)	47.3 (36.3)	0.8131
Premorbid BMI (kg/m²)	19.9 (2.91)	20.2 (2.83)	0.4515
AN-subtype [N(%)]:			0.3470
Binge-Purge	17 (21.8%)⁺	14 (15.1%)*	
Restrictive	61 (78.2%)⁺	79 (84.9%)*	
Diagnosis of affective disorder [N (%)]*:			0.4019
no	41 (61.2%)	56 (69.1%)	
yes	26 (38.8%)	25 (30.9%)	
BDI-II score	18.8 (10.7)	19.8 (10.1)	0.5116
BMI kg/m²	14.9 (1.40)	15.1 (1.28)	0.1766
BMI percentile	2.05 (4.84)	1.94 (3.18)	0.8588
BMI-SDS	-2.79 (1.06)	-2.67 (0.91)	0.4007
Vitamin D supple-mentation [N(%)]:			0.0728
no	25 (31.6%)	43 (46.2%)	
yes	54 (68.4%)	50 (53.8%)	

BDI-II = Beck Depression Inventory, AN= anorexia nervosa; ⁺information available for 78 patients; * information available for 81 patients, SDS = Standard Deviation Score; ANDI = Anorexia Nervosa Day patient versus Inpatient multicenter trial
